# Reactive Oxygen Species as a Response to Wounding: *In Vivo* Imaging in *Arabidopsis thaliana*

**DOI:** 10.3389/fpls.2019.01660

**Published:** 2020-01-09

**Authors:** Ankush Prasad, Michaela Sedlářová, Anastasiia Balukova, Marek Rác, Pavel Pospíšil

**Affiliations:** ^1^ Department of Biophysics, Centre of the Region Haná for Biotechnological and Agricultural Research, Faculty of Science, Palacký University, Olomouc, Czechia; ^2^ Department of Botany, Faculty of Science, Palacký University, Olomouc, Czechia

**Keywords:** Arabidopsis, confocal microscopy, fluorescent probes, mechanical injury, wounding

## Abstract

Mechanical injury or wounding in plants can be attributed to abiotic or/and biotic causes. Subsequent defense responses are either local, i.e. within or in the close vicinity of affected tissue, or systemic, i.e. at distant plant organs. Stress stimuli activate a plethora of early and late reactions, from electric signals induced within seconds upon injury, oxidative burst within minutes, and slightly slower changes in hormone levels or expression of defense-related genes, to later cell wall reinforcement by polysaccharides deposition, or accumulation of proteinase inhibitors and hydrolytic enzymes. In the current study, we focused on the production of reactive oxygen species (ROS) in wounded Arabidopsis leaves. Based on fluorescence imaging, we provide experimental evidence that ROS [superoxide anion radical (O_2_^•−^) and singlet oxygen (^1^O_2_)] are produced following wounding. As a consequence, oxidation of biomolecules is induced, predominantly of polyunsaturated fatty acid, which leads to the formation of reactive intermediate products and electronically excited species.

## Introduction

In biological systems, the metabolism is affected by non-physiological conditions which lead to stress reactions (Foyer et al., 1994; Cramer et al., 2011). The stress conditions in plants are categorized as biotic or abiotic; the former include herbivory, viral, bacterial, and fungal infections and damage by pests while the later include extreme environmental factors such as temperature, UV radiation, light, water availability, pH, salinity, toxic chemicals, burning, and mechanical injury among others (Garces et al., 2001; Johansson Jankanpaa et al., 2013; Kasai et al., 2019). The stressors can act independently or in various combinations (Savatin et al., 2014).

In plants, active i.e. biochemical defense responses have been well known to occur under the condition of wounding. Activation of local response to repair the damages occurs *via* stress-responsive gene, oxidative burst linked with cell wall reinforcement, deposition of callose, suberin, synthesis of various phenolics, defensive proteins, lectins, accumulation of phytoalexins etc. (Reymond et al., 2000; Savatin et al., 2014; Rehrig et al., 2014). The defense responses are known to be mediated by jasmonic acid, salicylic acid, abscisic acid, brassinosteroids, and strigolactones, ethylene (Verma et al., 2016). In Arabidopsis, several genes have been shown to be induced by wounding as summarized by Reymond and co-workers (Reymond and Farmer, 1998; Reymond et al., 2000). Plant response to wounding results from a complex network of physiological responses and depends on the nature of the threat and developmental stage of the plant (Taylor et al., 2004).

**Scheme 1:**
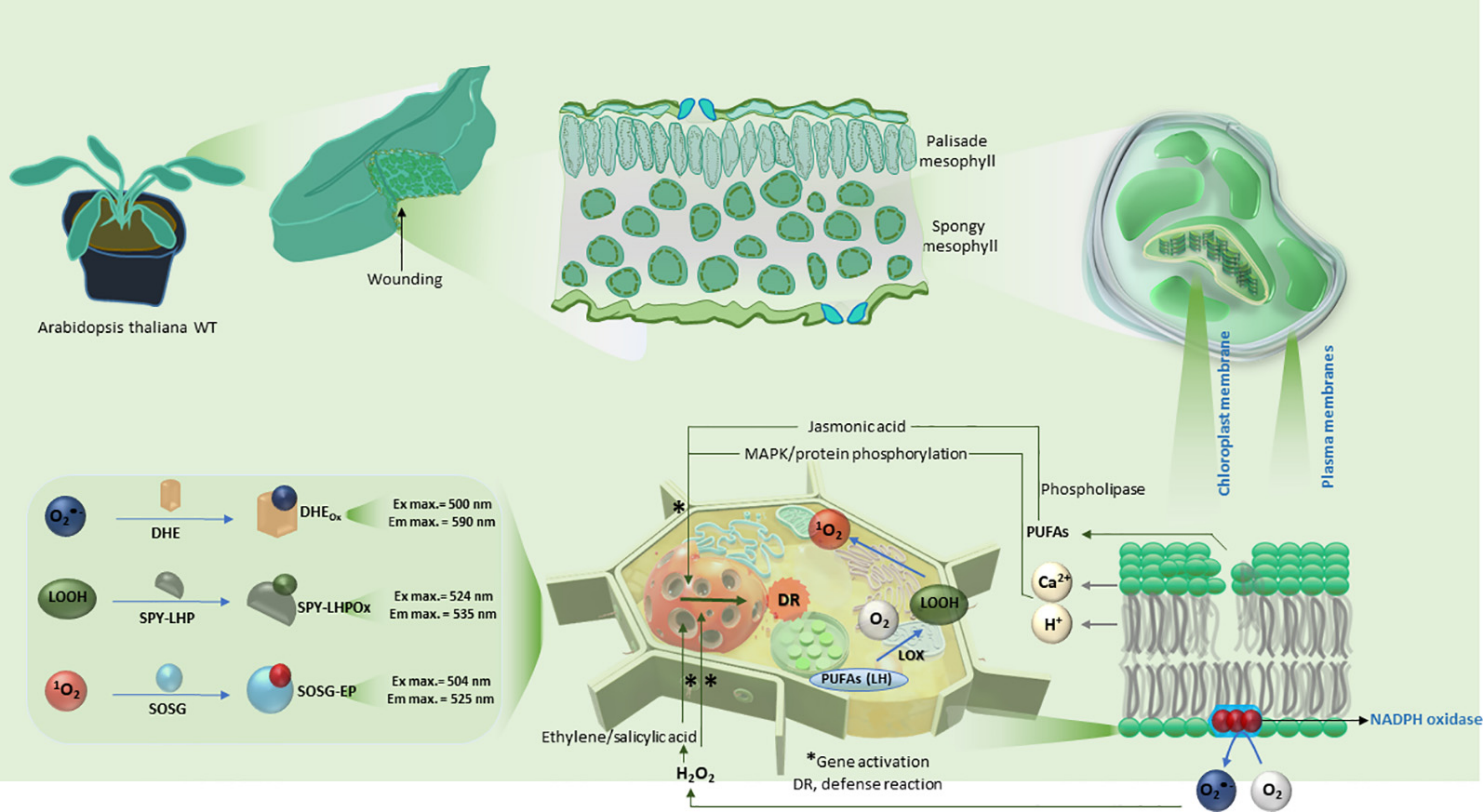
Schematic representation of the induction of mechanical injury in Arabidopsis leaves. The polyunsaturated fatty acids within the plasma and chloroplast membranes are shown to be oxidized in reactions subsequently leading to the formation of reactive oxygen species (O_2_^•−^ and ^1^O_2_) and LOOH. The lower panel shows a comprehensive summary of reaction mechanism leading to expression of defense response genes and details on usage of fluorescent probes utilized in confocal laser scanning microscopy.

In photosynthetic organism, generation of reactive oxygen species (ROS) is a quite universal and fast defense mechanism, known to be associated with various stresses both *in vivo* and *in vitro* (Yadav and Pospíšil, 2012; Prasad et al., 2015; Prasad et al., 2016; Prasad et al., 2017a; Prasad et al., 2018; Kumar et al., 2019), in both local as well as systemic responses (Grant and Loake, 2000; Slesak et al., 2007). In tomato, it has been observed that hydrogen peroxide (H_2_O_2_) was produced at the site within an hour of wounding and its level was enhanced even in distant part (upper unwounded leaves) in the following 4 to 6 h (Orozco-Cardenas and Ryan, 1999). The wounding of cells stimulates the influx of ions into the cytoplasm which in turn activates MAP kinases which are translocated into the nucleus thus activating genes involved in plant defense (**Scheme 1**). Furthermore, the influx of Ca^2+^ activates the production of superoxide anion radical (O_2_^•−^) by NADPH-dependent oxidase (**Scheme 1**). Under pathogen attack or following wounding, ROS have also been known to play a key role as signaling molecules (Mittler et al., 2011; Suzuki and Mittler, 2012).

Reactive oxygen species in high concentration can be toxic, therefore the plants have evolved an antioxidant system which includes the enzymatic and non-enzymatic antioxidant system (Foyer and Shigeoka, 2011; Bela et al., 2015). Under the circumstances, when production of ROS and antioxidant systems are in homeostasis, the ROS and intermediate products are known to act as signaling molecules (Waszczak et al., 2018; Kreslavski et al., 2012; Dietz et al., 2016; Czarnocka and Karpinski, 2018). The polyunsaturated fatty acids (PUFA’s) are the main target of ROS due to the presence of unsaturated double bonds. (Roach et al., 2015). The HO^•^ and the O_2_^•−^ are known to react with PUFA methylene groups leading to the formation of lipid alkyl and lipid peroxyl radicals, lipid hydroperoxides (LOOH), lipid alkoxyl radicals, and conjugated dienes (Smirnoff, 2000; Devasagayam et al., 2003; Saeidfirozeh et al., 2018). The peroxyl radicals are reactive intermediates and are known to be associated with the propagation part of the lipid peroxidation (Miyamoto et al., 2007). The lipid peroxidation in the biological membranes is the most obvious symptoms visible in plants as an outcome of oxidative stress in plants (Jarvis, 2011; Kumar et al., 2018; Zhang et al., 2018). The high energy intermediates (dioxetanes and tetroxide) formed during the oxidative radical reactions decompose to triplet carbonyls (^3^C=O*) which can then transfer triplet energy to molecular oxygen creating ^1^O_2_ (Di Mascio et al., 1992; Miyamoto et al., 2003; Miyamoto et al., 2007; Miyamoto and Di Mascio, 2014; Miyamoto et al., 2014; Cifra and Pospíšil, 2014; Pospíšil et al., 2019).

Various methods have been used for the detection of ROS and oxidative stress which include the use of electron paramagnetic resonance spectroscopy using various spin traps and spin probes, optical spectroscopy using fluorescent and chemiluminescent probes, electrochemical biosensors, chromatography etc. (Zhang et al., 2018). Within the frame of the current study, we have attempted to visualize the formation of O_2_^•−^, LOOH, and ^1^O_2_ as a result of the mechanical injury in Arabidopsis leaves using confocal laser scanning microscopy.

## Materials and Methods

### Fluorescent Probes and Chemical Reagents

Fluorescent probe, dihydroxyethidium (DHE) was purchased from Sigma Aldrich GmbH (Germany); Spy-LHP from Dojindo Molecular Technologies Inc. (Rockville, MD, USA) and Singlet Oxygen Sensor Green (SOSG) from Molecular Probes Inc. (Eugene, OR, USA). All other chemicals of analytical grade were purchased from Sigma Aldrich GmbH (Germany).

### Arabidopsis Plants

*Arabidopsis thaliana* WT (Columbia-0) was obtained from the Nottingham Arabidopsis Stock Centre (NASC), University of Nottingham (Loughborough, United Kingdom). Plants were grown in Fytoscope FS-WI-HY (Photon Systems Instruments, Drásov, Czech Republic) using a peat substrate (Klasmann, Potground H) following 4 days of soaking of seeds in distilled water. The plants were grown 6 weeks under the following conditions: photoperiod of 8/16 h light/dark; photon flux density 100 µmol photons m^-1^ s^-1^; temperature: 22˚/20˚C light/dark and relative humidity: 60%.

### Sample Preparation and Confocal Laser Scanning Microscopy

**Figure 1:**
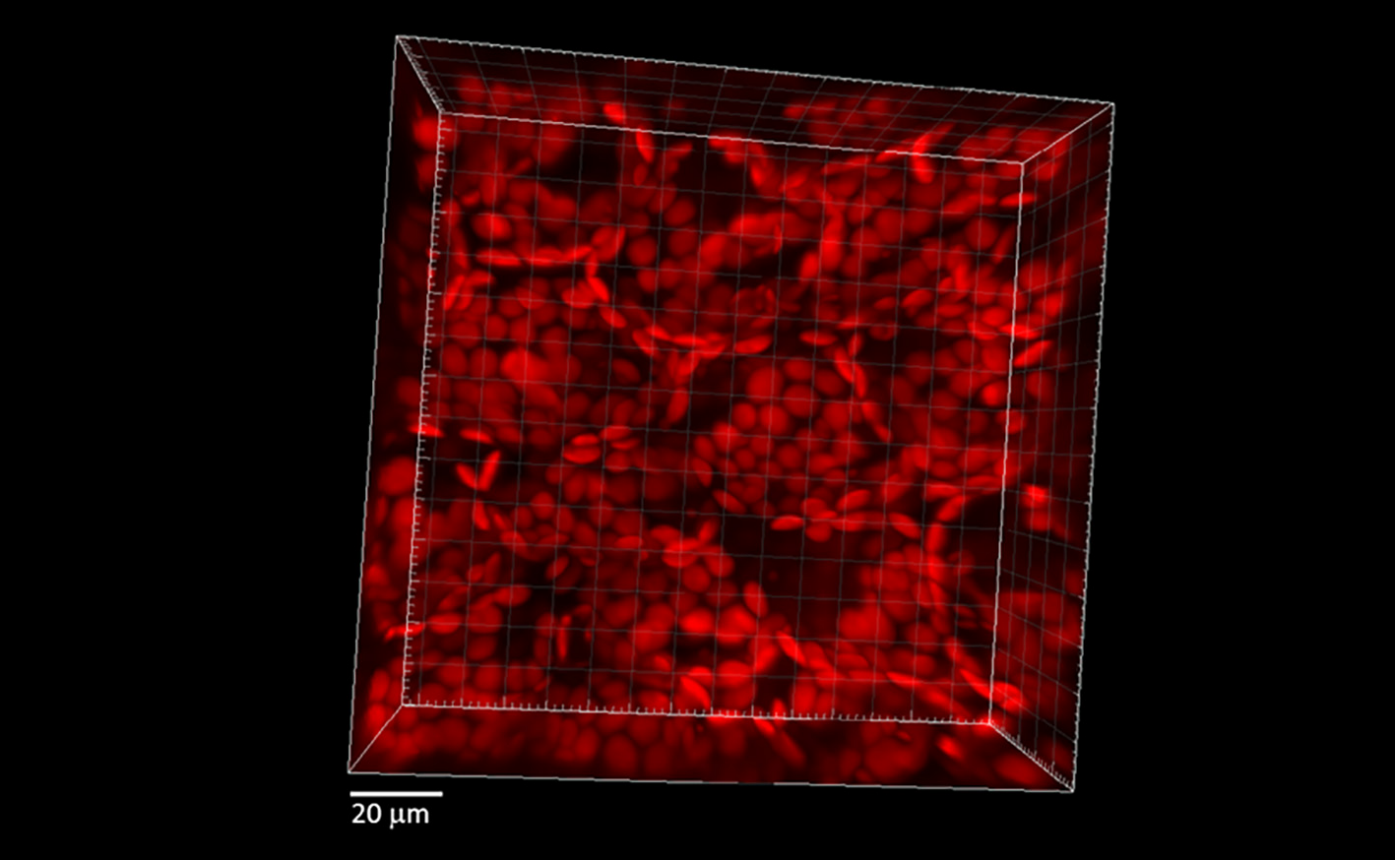
Spatial distribution of chloroplasts within mesophyll of Arabidopsis leaves kept under diffused green light for 90 min prior to imaging. The 2.5 projection represents a series of forty optical sections (x, y) sequentially acquired at z  =  1 μm.

Mechanical injury of Arabidopsis leaves was carried out using a sharp razor blade. Leaf pieces of ca 5 × 5 mm were cut out in HEPES buffer (pH 7.5) and infiltrated with fluorochromes using a syringe (see protocol in references) (Kumar et al., 2018; Prasad et al., 2018). Following 30 min incubation in desired probe (100μM/250μM DHE, 50 μM Spy-LHP, or 50 μM SOSG), tissues were transferred into HEPES buffer on a glass slide and visualized by Fluorview 1000 confocal laser scanning microscope (Olympus Czech Group, Prague, Czech Republic). The arrangement of chloroplasts within the mesophyll cells was visualized (**Figure 1**). The image shows the spatial distribution of chloroplasts within mesophyll of Arabidopsis leaves kept under diffused green light for 90 min prior to imaging. The 2.5 projection represents a series of forty optical sections (x, y) sequentially acquired at z  =  1 μm. It can be observed that the chloroplasts are distributed on the cell surface which is a characteristic behavior under weak light conditions to absorb more light (Jarvis, 2011).

**Figure 2:**
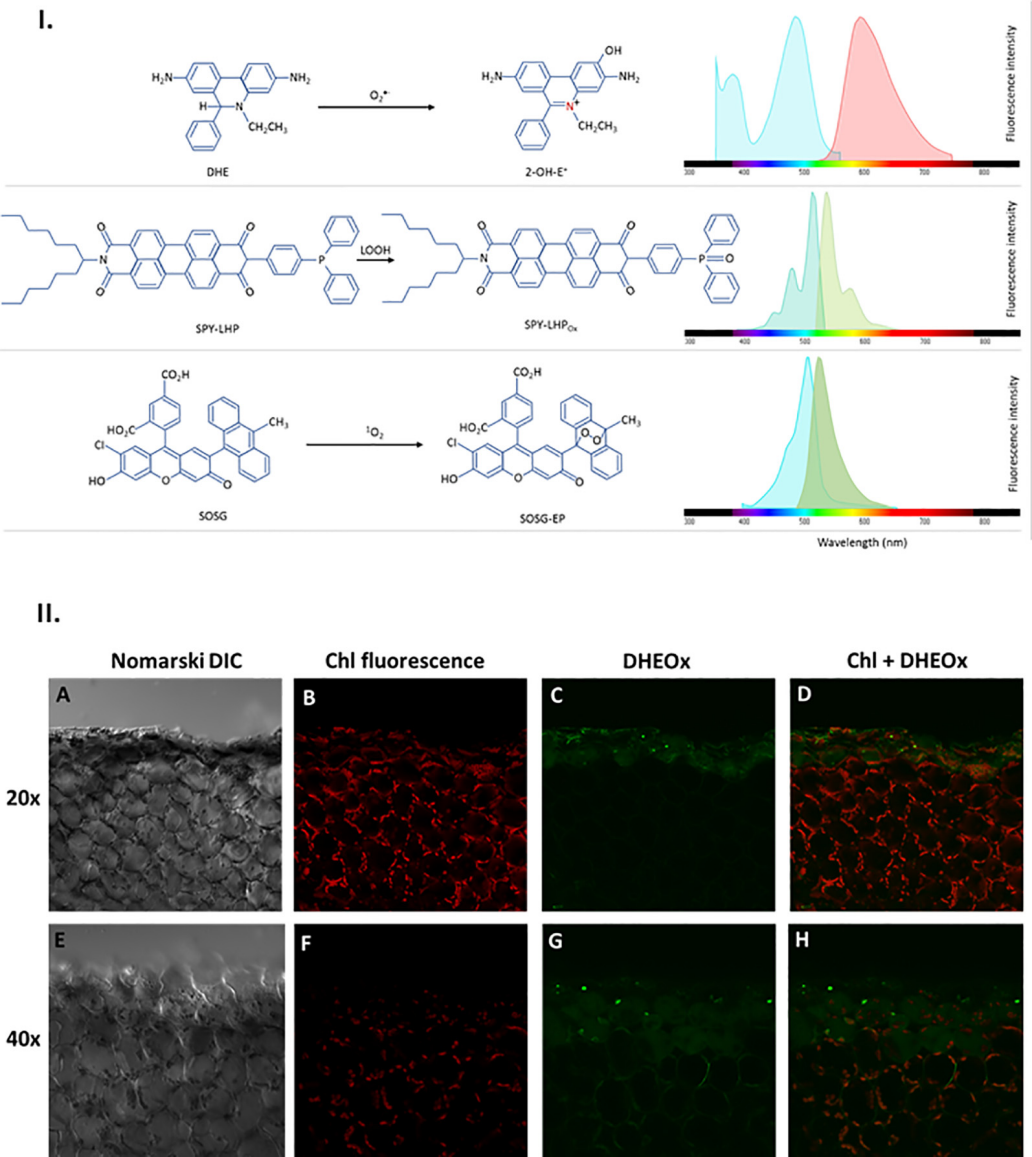
**I.** Principles of ROS detection and fluorochrome spectral properties **(A)** DHE oxidation by O_2_^•−^ forming 2-OH-E^+^ providing fluorescence with excitation/emission maxima of ~500/590 nm. **(B)** SPY-LHP oxidation by LOOH forming SPY-LHPOx providing fluorescence with excitation/emission maxima of ~524/535 nm. **(C)** SOSG oxidation by ^1^O_2_ forming fluorescent SOSG-EP with excitation/emission maxima of ~504/525 nm). **II**. Superoxide anion radical imaging in cells of WT Arabidopsis leaves detected by confocal laser scanning microscope. The panels (from left to right) represents the Nomarski DIC **(A, E)** chl fluorescence **(B, F)** DHEox fluorescence **(C, G)** and combined (chl fluo + DHEox) **(D, H)** channel following 30 min of incubation in DHE [100 μM (upper panel)/250 μM (lower panel)] in the presence of 0.01% DMSO. The margins indicate the site of mechanical injury visualized under objective of of 20× (upper panel) and 40× (lower panel). The fluorescence signal was visualized with an excitation (λex) and emission (λem) wavelengths of 488 nm and 505–605 nm respectively. Chloroplasts imaging was achieved with laser excitation at 543 nm and emission recorded at 655–755 nm.

The excitation of DHE, SPY, and SOSG was performed using a 488 nm line of an argon laser and the emission was detected by a 505–605 nm filter for DHE, 505–550 nm filter for Spy-LHP and 505–525 nm filter for SOSG (**Figure 2I**). The cell morphology was visualized using 405 nm diode laser excitation by transmitted light detection module and differential interference contrast (DIC) filters. Chloroplasts were visualized based on autofluorescence of photosynthetic pigments with excitation by a 543 nm helium-neon laser, and emission recorded with a 655–755 nm bandpass filter. The proper intensity of lasers was set according to unstained samples at the start of each experiment (Sedlarova et al., 2011). All confocal experiments were done in several replicates and the representative images have been presented.

### Measurement Setup and Charge Coupled Device Imaging

It is imperative to control any kind of interference from the stray photons and thus a dark room specifically designed for measuring ultra-weak photon emission as described in our previous studies was utilized (Prasad and Pospíšil, 2013). The ultra-weak photon emission imaging was performed using a highly sensitive CCD camera VersArray 1300B (Princeton Instruments, Trenton, NJ, USA). The CCD camera was cooled down to −110°C using a liquid-nitrogen cooling system to reduce the dark current. The measurement was done in the image format of 1,340 × 1,300 pixels and the data correction was done by subtracting the background prior to measurement. Other experimental conditions were: spectral sensitivity, 350 to 1,000 nm; readout speed, 100 kHz; gain, 2; and accumulation time: 20 min. All other settings were as in Prasad and Pospíšil (Prasad and Pospíšil, 2013). In order to avoid any kind of intervention of delayed luminescence, the Arabidopsis plant was dark-incubated for approximately 2 h. The mechanical injury was induced using a sharp blade in the presence of diffused green light with precaution not to exert any external mechanical pressure on other parts of the Arabidopsis plant/leaves. The data accumulation was started 20 min after the mechanical injury. All measurements were done in at least three replicates and the representative images have been presented.

## Results and Discussion

### Wounding and Superoxide Anion Radical Imaging

The O_2_^•−^ formation in mechanically injured Arabidopsis leaves was studied using the fluorescent probe, DHE using confocal laser scanning microscopy. **Figure 2II** and **Supplementary Data 1** represent Nomarski DIC [1II (A), (E) and S1(A)], chlorophyll fluorescence [1II (B), (F) and S1(B)], DHEOx fluorescence [1II (C), G and S1(C)], and a merge of chlorophyll and DHEOx fluorescence channel images [1II (D), (H) and S1(D)] measured in a mechanically injured Arabidopsis leaf. The presented result shows that there is a formation of O_2_^•−^ on the cut edge of Arabidopsis leaves [1II (C) and 1II (G)]. It can be clearly observed that in cells succeeding the mechanical injury, the DHEOx fluorescence is from the cellular volume of the cells which apparently includes the chloroplast, plasma membrane, and the cytoplasm. It is believed that in these cells, there is an overall higher impact due to mechanical injury. Images of the merged channels depict that a high extent of both chloroplast/plasma membrane integrity is maintained, however, some non-visible disturbance in the cellular integrity can be taken into consideration which might have resulted in DHEOx fluorescence observed in the cytoplasm [1II (C) and 1II (G)]. In addition to this, it can also be hypothesized that the DHEOx fluorescence observed in the cytoplasm can be a consequence of potential diffusion of O_2_^•−^ because of the porous membrane formed as a result of the mechanical injury. To validate the production of O_2_^•−^, the effect of superoxide dismutase (SOD), which leads to the dismutation of O_2_^•−^ to H_2_O_2_ on ultra-weak photon emission was tested and has been described later.

Dihydroxyethidium is a widely used ethidium-based, redox-sensitive fluorescent probe which is known to passively diffuse into cells and commonly used to detect cytosolic O_2_^•−^ (Wojtala et al., 2014a). It has been shown to be oxidized by O_2_^•−^ to form 2-hydroxyethidium (2-OH-E^+^) emitting at 590 nm [**Figure 2I**] (Zielonka and Kalyanaraman, 2010). Histochemical staining using nitroblue tetrazolium (NBT) was used in the past to detect O_2_^•−^ in wounded leaves which is in agreement with our study showing the formation of O_2_^•−^ in and around the vicinity of the mechanically injured site (Wohlgemuth et al., 2002; Morker and Roberts, 2011). Dihydroxyethidium has been the most commonly used fluorescent probe for the detection of O_2_^•−^ although it was shown to undergo unspecific oxidation by ONOO- or HO^•^ into ethidium (Wojtala et al., 2014b). Therefore, it is highly recommended that precise control experiments should be performed to avoid any misinterpreting of results.

### Wounding and Lipid Hydroperoxide Imaging

**Figure 3:**
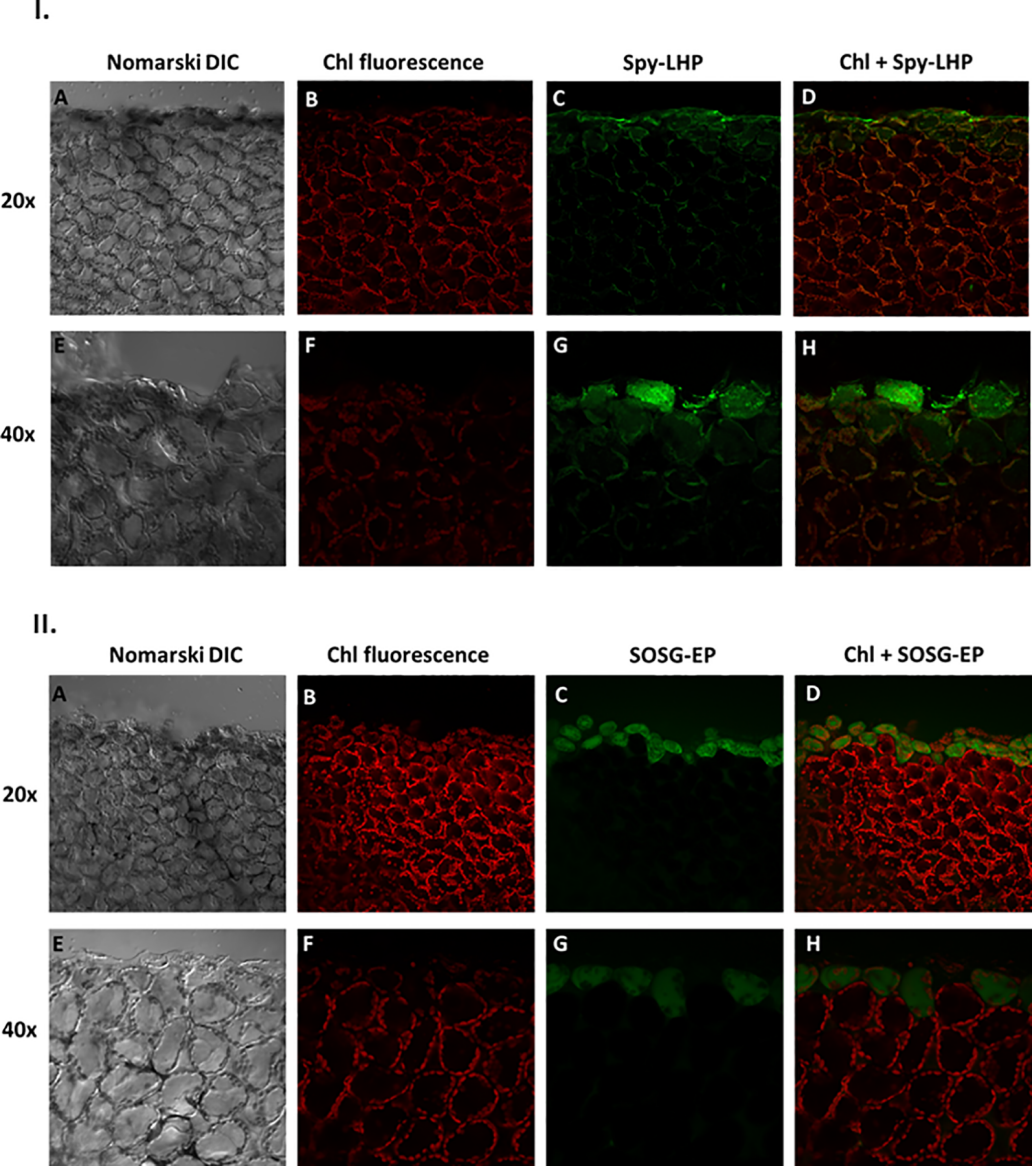
**I.** Imaging of lipid hydroperoxide within Arabidopsis leaves by confocal microscopy. The panels (from left to right) represent DIC **(A, E)**; chlorophyll fluorescence **(B, F)**; Spy-LHPOx fluorescence **(C, G)** and combined channel (chl fluorescence + Spy-LHPOx) **(D, H)** following 30 min of incubation in 50 μM Spy-LHP under objective of 20× (upper panel) or 40× (lower panel). The formation of LOOH was measured in Arabidopsis leaves with excitation (λex) and emission (λem) wavelength of 488 nm and 505–550 nm, respectively. **II.** Confocal microscopy imaging of singlet oxygen formed during mechanical injury of Arabidopsis leaves. The panels represent (from left to right): DIC **(A, E)**; chl fluorescence **(B, F)** SOSG-EP fluorescence **(C, G)** and combined channel (chl fluorescence + SOSG-EP) **(D, H)** under 20× and 40× objective following 30 min of incubation in 50 μM SOSG. The SOSG-EP fluorescence was excited by 488 nm and emission recorded at 505–525 nm.

The LOOH formation in mechanically injured Arabidopsis leaves was monitored using a fluorescent probe, Spy-LHP. Spy-LHP is a swallow-tailed perylene derivative predominately used for live cell imaging of phospholipid peroxide (**Figure 2I**) (Soh et al., 2006; Soh et al., 2007). Spy-LHP is highly selective to LOOH and does not react with H_2_O_2_, HO^•^, O_2_^•−^, nitric oxides, peroxynitrite, and peroxyl radicals. **Figure 3I** shows Nomarski DIC [3I(A) and 3I(E)], chlorophyll fluorescence [3I (B) and 3I (F)], Spy-LHPOx fluorescence [3I (C) and 3I (G)], and a merge of chlorophyll and Spy-LHPOx fluorescence channel images [3I (D) and 3I (H)] measured in Arabidopsis leaves. The observation that the localization of chlorophyll fluorescence overlaps precisely with the localization of Spy-LHPOx fluorescence confirms that LOOH is formed mostly in chloroplasts [3I (D) and 3I (H)]. It can also be clearly seen that only one layer of the cells at the cut edge of the leaf has a brighter green fluorescence in comparison to DHEOx fluorescence where a few adjoining cell layers show the fluorescence signal from cellular volume (**Figures 2II**, **3I**, and **Supplementary Data 1**). Hence, it can be stated here that Spy-LHPOx fluorescence signal is even more localized close to the site of mechanical injury which can be justified by the fact that LOOH is comparatively larger intermediates compared to O_2_^•−^. Our results presented on Arabidopsis leaves/Chlamydomonas cells with the employment of lipoxygenase mutant/use of inhibitors of lipid peroxidation also favors the conclusion that lipid hydroperoxide formation is prevalent in photosynthetic samples (**Supplementary Data 2**) (Prasad and Pospíšil, 2011; Prasad et al., 2017b).

Spy-LHP was developed by Soh and co-workers as a fluorescent probe for the detection of live-cell imaging of lipid hydroperoxide (Soh et al., 2006). It has been one of the most widely used probe for this purpose, mostly in photosynthetic cells. We have recently tested Spy-LHP usability for detection of protein hydroperoxide (Pathak et al., 2017); however, its selectivity in presence of protein and lipid hydroperoxides mixture is presumed to be inclined toward the lipid hydroperoxides. Spy-LHP is still regarded as the most appropriate probe which should be considered for investigating the LOOH. However, it suffers from the limitation due to its limited solubility in a low cytotoxic organic solvent such as ethanol and dimethyl sulfoxide (DMSO) and high hydrophobicity (Yamanaka et al., 2012).

### Wounding and Singlet Oxygen Imaging

To visualize the ^1^O_2_ formation in the mechanically injured leaves of Arabidopsis leaves, the fluorescent probe, SOSG was used. Singlet Oxygen Sensor Green is known for its high selective properties for ^1^O_2_ and does not show any appreciable response to HO^•^ or O_2_^•−^. Under normal conditions, SOSG exhibits weak blue fluorescence, but in the presence of ^1^O_2_, it emits green fluorescence with the maximum wavelength at 525 nm (**Figure 2I**). **Figure 3II** demonstrates the Nomarski DIC images [3II (A) and 3II (E)], the chlorophyll fluorescence [3II (B) and 3II (F)], the SOSG endoperoxide (SOSG-EP) fluorescence [3II (C) and 3II (G)], and the merge of chlorophyll and SOSG-EP fluorescence channels images [3II (D) and 3II (H)] measured in mechanically injured Arabidopsis leaves. It can be clearly observed that the signals from both channels overlap. It is evident that ^1^O_2_ has limitation pertaining to diffusion which can be because of its shorter half-life; however, it is well known to bear signaling role (Kochevar, 2004; Kim et al., 2008; Triantaphylides and Havaux, 2009) mediated *via* the local and systemic responses. Our results were further validated using ultra-weak photon emission imaging in the presence of ^1^O_2_ scavenger histidine and has been described later.

Singlet Oxygen Sensor Green has been used during the past decade and has faced criticism predominantly when used under exogenous light illumination (Hideg, 2008; Ragas et al., 2009). However, recently we have discussed (Sedlarova and Luhova, 2017) and presented a comprehensive study on the limitation associated with its usage for sensitive and selective detection of ^1^O_2_ (Prasad et al., 2018). Nevertheless, considering the concerns, it is critical to combine methods especially when *in-vivo/ex-vivo* experiments utilizing fluorescent probes are performed since biological systems have complex cellular environment where actual redox state can interfere with the ongoing signal leading to false positive result or cross-sensitivity to cellular antioxidants that compete with the ROS probes thereby leading to false negative results (Ortega-Villasante et al., 2018).

### Wounding, Oxidative Radical Reaction, and Ultra-Weak Photon Emission

**Figure 4:**
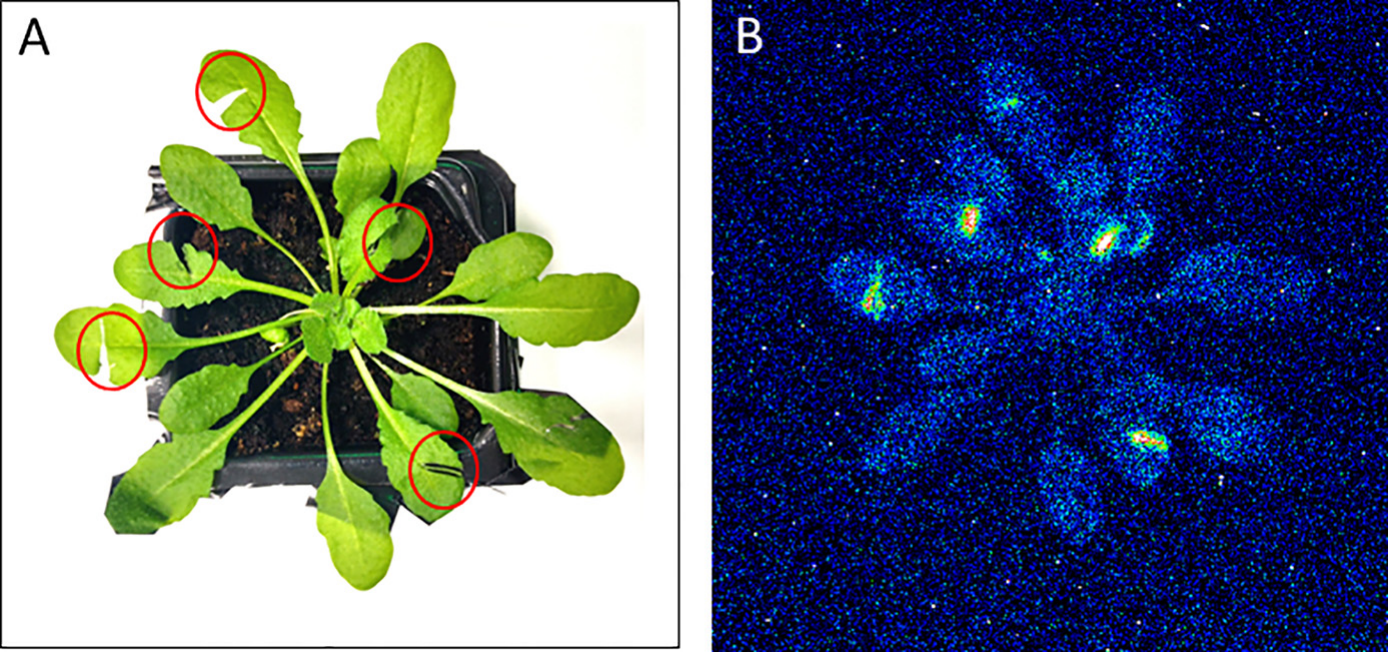
Two-dimensional imaging of the ultra-weak photon emission from the plant of *Arabidopsis thaliana*. The figure shows photographs **(A)** and the corresponding two-dimensional images of ultra-weak photon emission recorded by a highly sensitive CCD camera **(B)**. The Arabidopsis plant was kept in the complete darkness for a period of 2h prior to the measurement. Ultra-weak photon emission imaging was measured 20 min after wounding (indicated by red circles) with an accumulation time of 20 min.

**Figure 5:**
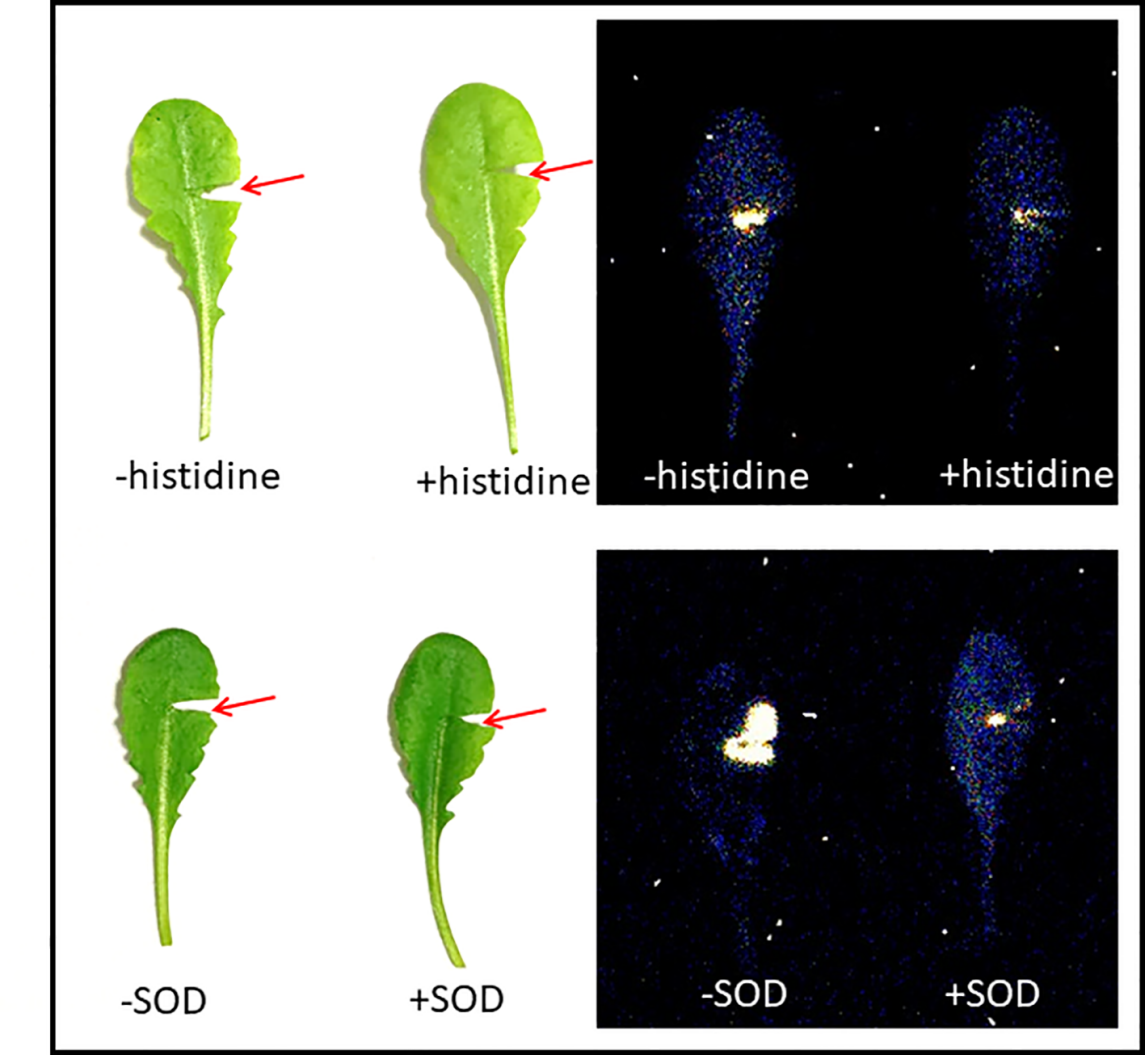
Ultra-weak photon emission imaging measured in mechanical injured Arabidopsis leaves in the absence and presence of 10 mM histidine (upper panel) and 400U/ml superoxide dismutase (SOD) (lower panel). The integration time of 30 min was kept and all other experimental conditions as in **Figure 4**.

Mechanical injury in Arabidopsis is known to generate triplet excited carbonyls (^3^C=O^*^) through induction of oxidative radical reactions. **Figure 4** shows a photograph (A) and two-dimensional imaging of ultra-weak photon emission measured in Arabidopsis plant. The parts of the Arabidopsis leaves marked with a red circle indicates the mechanical injury. It can be clearly seen that ultra-weak photon emission was considerably enhanced as compared to non-wounded parts of the leaves. Based on the spatial distribution, higher intensity of ultra-weak photon emission is only prevalent at the injured site leading to the conclusion that the oxidative radical reaction is restricted only to the site of mechanical injury which is in good agreement with the presented data about ROS formation from confocal laser scanning microscopy. It can be suggested that the ROS are generated as a consequence of wounding in Arabidopsis leaves. The overall photon emission observed at the site of injury can be attributed to ROS produced and consecutive oxidative radical reactions which led to the formation of electronically excited species. The ultra-weak photon emission observed in **Figure 4** can be attributed to the emission from ^3^C=O^*^ and singlet chlorophylls (^1^Chl*) formed from excitation energy transfer from ^3^C=O^*^ to chlorophylls and/or ^1^O_2_ dimol emission (**Scheme 1**) (Prasad et al., 2017). The results were validated using histidine and SOD (**Figure 5**).

Several challenges must be overcome during the usage of fluorescent probes in confocal laser scanning microscopy which include the short half-life of ROS, cross-reactivity of fluorescent probes, uneven uptake of probes by cells within tissues, dose-dependent toxicity (either of the fluorescent compounds and/or solvent). Besides problems, the use of fluorescent probes is among the best methods to sensitively and selectively identify the reactive species and intermediates. Since the ROS and related redox changes modulate the signaling event, the use of fluorescent probes is considered beneficial in understanding signaling in plants.

## Data Availability Statement

All datasets generated for this study are included in the article/**Supplementary Material**.

## Author Contributions

AP and PP contributed to the conception of the work. AP and MS performed the measurements and AB assisted in measurements. AP analyzed, interpreted the data and wrote the manuscript. AB participated in the drafting of the first version of the manuscript. MR contributed in the standardization of fluorescent probes utilized. PP and MS revised it critically for important content. All authors approved the final version of the manuscript.

## Funding

This work was financially supported by the European Regional Development Fund (ERDF) project “Plants as a tool for sustainable global development” (no. CZ.02.1.01/0.0/0.0/16_019/0000827) and Internal Grant Agency of Palacký University (grant nos. IGA_PrF_2019_030 and IGA_PrF_2019_004).

## Conflict of Interest

The authors declare that the research was conducted in the absence of any commercial or financial relationships that could be construed as a potential conflict of interest.

## Abbreviations

ROS, reactive oxygen species; CCD, charge-coupled device; O_2_^•−^, superoxide anion radical; LOOH, lipid hydroperoxide; ^1^O_2_, singlet oxygen.
