# Supplementary material for: Reactive Oxygen Species as a Response to Wounding: In Vivo Imaging in Arabidopsis thaliana
Source: Front Plant Sci. 2020 Jan 9;10:1660. doi: 10.3389/fpls.2019.01660 (PMC6962234; doi:10.3389/fpls.2019.01660)
Supplement: Supplementary file 1 [file DataSheet_1.pdf]

Supplementary data 1

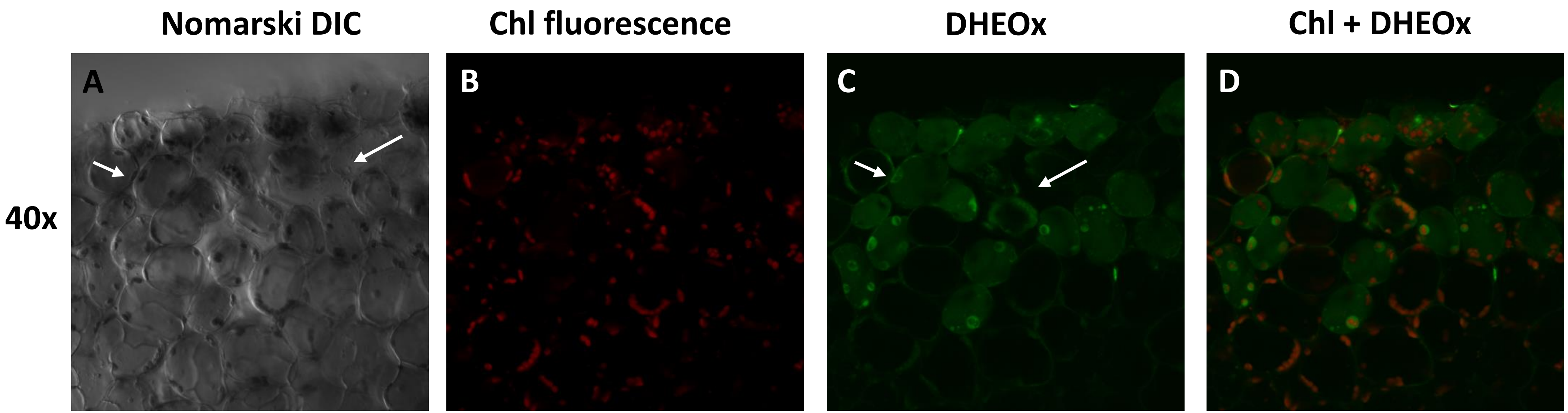

Supplementary data 1: Superoxide anion radical imaging in cells of WT Arabidopsis leaves detected by confocal laser scanning microscope. The panels (from left to right) represents the Nomarski DIC; chl fluorescence; DHEox fluorescence and combined (chl fluo + DHEox) channel following 30 min of incubation in DHE [250 μM] in the presence of 0.01% DMSO. The arrows indicate the sites of mechanical injury ~~measured at a magnification of~~ 40x. More cell layers are can be seen to be impacted (marked by arrows) in comparison to Fig. 1II. The fluorescence signal was measured with an excitation (λex) and emission (λem) wavelengths of 488 nm and 505-605nm respectively.
