# Supplementary material for: Reactive Oxygen Species as a Response to Wounding: In Vivo Imaging in Arabidopsis thaliana
Source: Front Plant Sci. 2020 Jan 9;10:1660. doi: 10.3389/fpls.2019.01660 (PMC6962234; doi:10.3389/fpls.2019.01660)
Supplement: Supplementary file 2 [file DataSheet_2.pdf]

Supplementary data 2

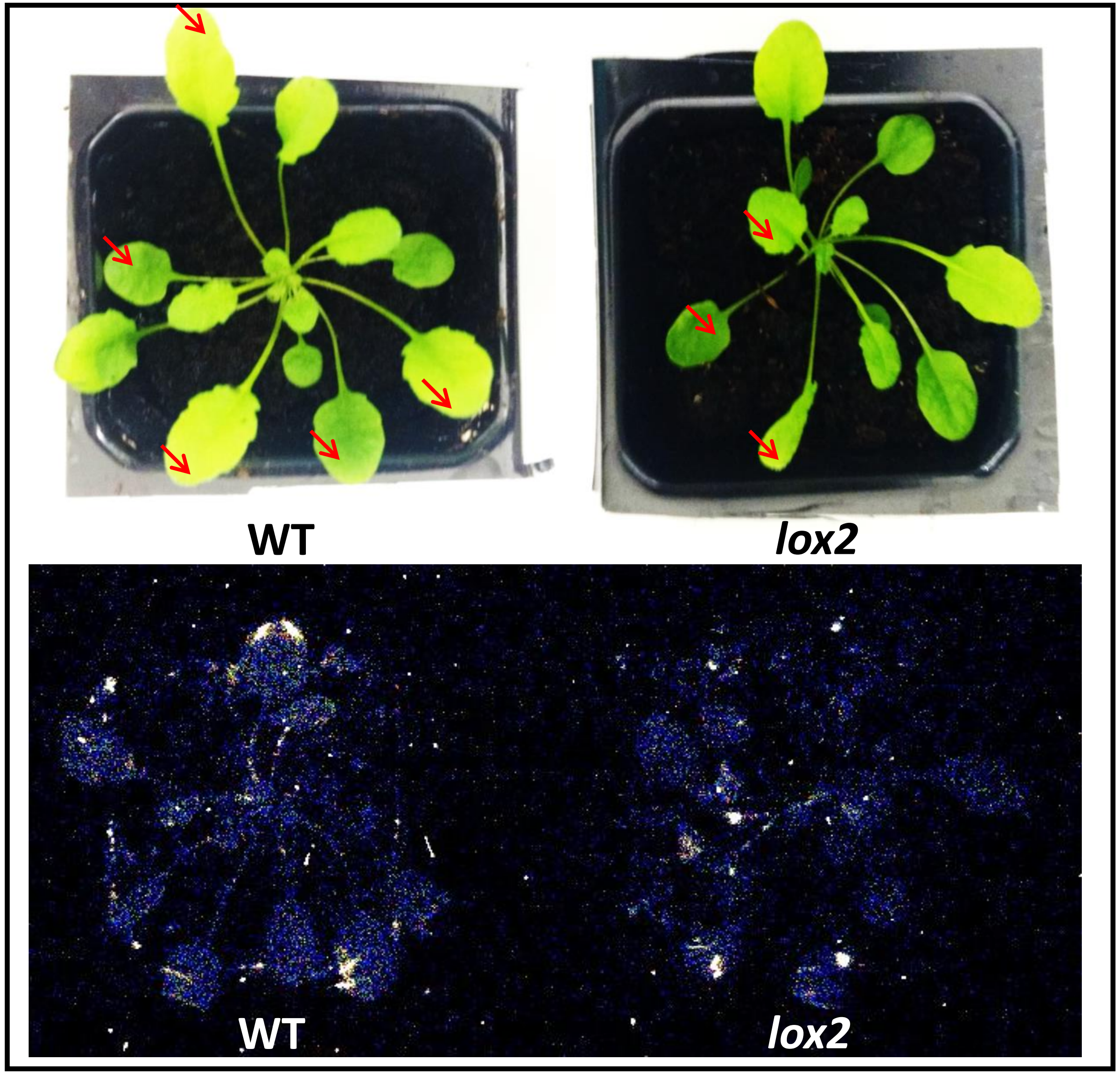

Supplementary data 2: Two-dimensional imaging of the ultra-weak photon emission from the *Arabidopsis thaliana*. The photographs (upper panel) and the corresponding two-dimensional images of spontaneous ultra-weak photon emission (lower panel). Ultra-weak photon emission imaging was measured in WT Arabidopsis plant and *lox2* mutant with an integration time of 30 min. The arrows in red indicate the mechanically injured part of the leaves.
